# Estimation of ventilatory thresholds during exercise using respiratory wearable sensors

**DOI:** 10.1038/s41746-024-01191-9

**Published:** 2024-07-26

**Authors:** Felipe Contreras-Briceño, Jorge Cancino, Maximiliano Espinosa-Ramírez, Gonzalo Fernández, Vader Johnson, Daniel E. Hurtado

**Affiliations:** 1 Laboratory of Exercise Physiology, Faculty of Medicine, Pontificia Universidad Católica de Chile, Santiago, Chile; 2 Laboratory of Exercise Physiology & Metabolism, Faculty of Medicine, Universidad Finis Terrae, Santiago, Chile; 3 Clínica Alemana de Santiago, Santiago, Chile; 4 IC Innovations SpA, Santiago, Chile; 5 Department of Structural and Geotechnical Engineering, School of Engineering, Pontificia Universidad Católica de Chile, Santiago, Chile; 6 Institute for Biological and Medical Engineering, Schools of Engineering, Medicine, and Biological Sciences, Pontificia Universidad Católica de Chile, Santiago, Chile; 7 Institute for Medical Engineering and Science, Massachusetts Institute of Technology, Cambridge, MA USA

**Keywords:** Health services, Quality of life

## Abstract

Ventilatory thresholds (VTs) are key physiological parameters used to evaluate physical performance and determine aerobic and anaerobic transitions during exercise. Current assessment of these parameters requires ergospirometry, limiting evaluation to laboratory or clinical settings. In this work, we introduce a wearable respiratory system that continuously tracks breathing during exercise and estimates VTs during ramp tests. We validate the respiratory rate and VTs predictions in 17 healthy adults using ergospirometry analysis. In addition, we use the wearable system to evaluate VTs in 107 recreational athletes during ramp tests outside the laboratory and show that the mean population values agree with physiological variables traditionally used to exercise prescription. We envision that respiratory wearables can be useful in determining aerobic and anaerobic parameters with promising applications in health telemonitoring and human performance.

## Introduction

Cardiopulmonary exercise testing (CPET) is the *gold-standard* for the evaluation of the cardiovascular, respiratory, and skeletal muscle systems during physical effort^1–3^. CPET has extensive applications in medicine, as it measures cardiometabolic and ventilatory markers that support the functional assessment of patients with heart failure^4,5^, coronary artery disease^6^, and pulmonary hypertension^7^, among others. CPET also plays a crucial role in sports sciences and professional training, as it provides metabolic information that enables the determination of ventilatory thresholds (VTs), key parameters in the prescription of exercise, and performance tracking of athletes^8,9^. While the impact of CPET in medicine and sports sciences is broad and well established, it requires a technical infrastructure and operation that restricts its use to the clinical and research settings^10^. This limitation, together with recent advances in physiological wearable sensors, represents a unique opportunity to create new wearable technology to assess cardiopulmonary performance at lower cost and outside complex facilities^11,12^.

VTs are physiological parameters widely used for the study and monitoring of physical fitness that are identified by the CPET software and subsequently corroborated by visual analysis by expert physiologists (*gold-standard*). The first ventilatory threshold (VT1), originally defined by Wasserman and McIlroy as anaerobic threshold (AT)^13^, denotes the point during exercise where ventilation increases faster than oxygen consumption (VO_2_). It also manifests as the first non-linear increase in MV during graded exercise tests^14^. The second ventilatory threshold (VT2), or also named as second AT or respiratory compensation point^15^, occurs during high exercise intensity, triggered by the overactivation of anaerobic metabolism needed to supply the energy demands. VT2 is observed during CPET as a secondary non-linear increase in MV, which concurrently arises with an increase in the carbon-dioxide ventilatory equivalent. These ventilatory changes are a consequence of metabolic compensations that are typically assessed from lactate tests^9,16^, which require blood sample collection that may not be compatible with mass testing or in-field tracking.

Based on VTs, Skinner and McLellan^17^ proposed a model compound of three phases (triphasic model) traditionally utilized by fitness coaches to prescribe exercise, considering the values’ range at which variables associated with physical effort are linked to VTs (i.e., heart rate (HR), blood lactate ([Lac], power, speed, etc.). Of these, HR is the most used, which shows a lineal increase as exercise progresses. In healthy subjects are expected to achieve a range of 130–150 beats·min^-1^ and 160–180 beats·min^-1^ in VT1 and VT2, respectively. Habitually, to improve cardiopulmonary exercise capacity subjects should complete a training where the first 4 weeks exercised at HR linked to VT1, from five to 8 weeks tailored at HR between VT1 and VT2, and finally the last three to 4 weeks train at HR above linked to VT2^18^. However, these HR ranges vary in clinical contexts depending on the population studied^19^; as well as in extreme environmental conditions (i.e., altitude^20^ and hypoxia^21^) where HR is not a good indicator of the level of exercise intensity, motivating the exploration of other physiological variables involved in identifying VTs.

Currently, a handful of sensor technologies and advanced algorithms are being explored in the estimation of VT1 and VT2. Using electrocardiography (ECG) signals acquired from commercially available systems, the time instant when AT occurs has been associated with the dynamics of the fractal correlation index of heart rate variability (HRV) during incremental treadmill tests in a group of healthy subjects^22^. Recent studies have also considered HRV features extracted from single-lead ECG signals in the construction of neural-network classifiers to detect VT1, using data from a cohort of 260 patients with cardiovascular disease undertaking CPET at a hospital facility^23^. For the determination of VT2, solutions based on near-infrared spectroscopy (NIRS) signals from portable devices have reported high agreement with physiological data from *gold-standard*^24^. While these technologies offer promising alternatives, most of them require medical-grade sensors and equipment that restrict their application to clinical and research laboratories. Further, systems based on data-driven algorithms carry the disadvantage of being predictive only for the population they were trained for^25^. To our knowledge, there is no single wearable technology able to provide accurate VT1 and VT2 estimations.

In this study, we validate a respiratory wearable system and algorithms for estimating VTs during cycling-graded exercise tests in healthy subjects. Additionally, we assess the applicability of our system outside laboratory facilities by evaluating VTs from ramp tests on smart trainers in recreational athletes, demonstrating concordance with classical physiological variables commonly used for exercise prescription.

## Results

### Validation of respiratory rate measurements during exercise

**Fig. 1:**
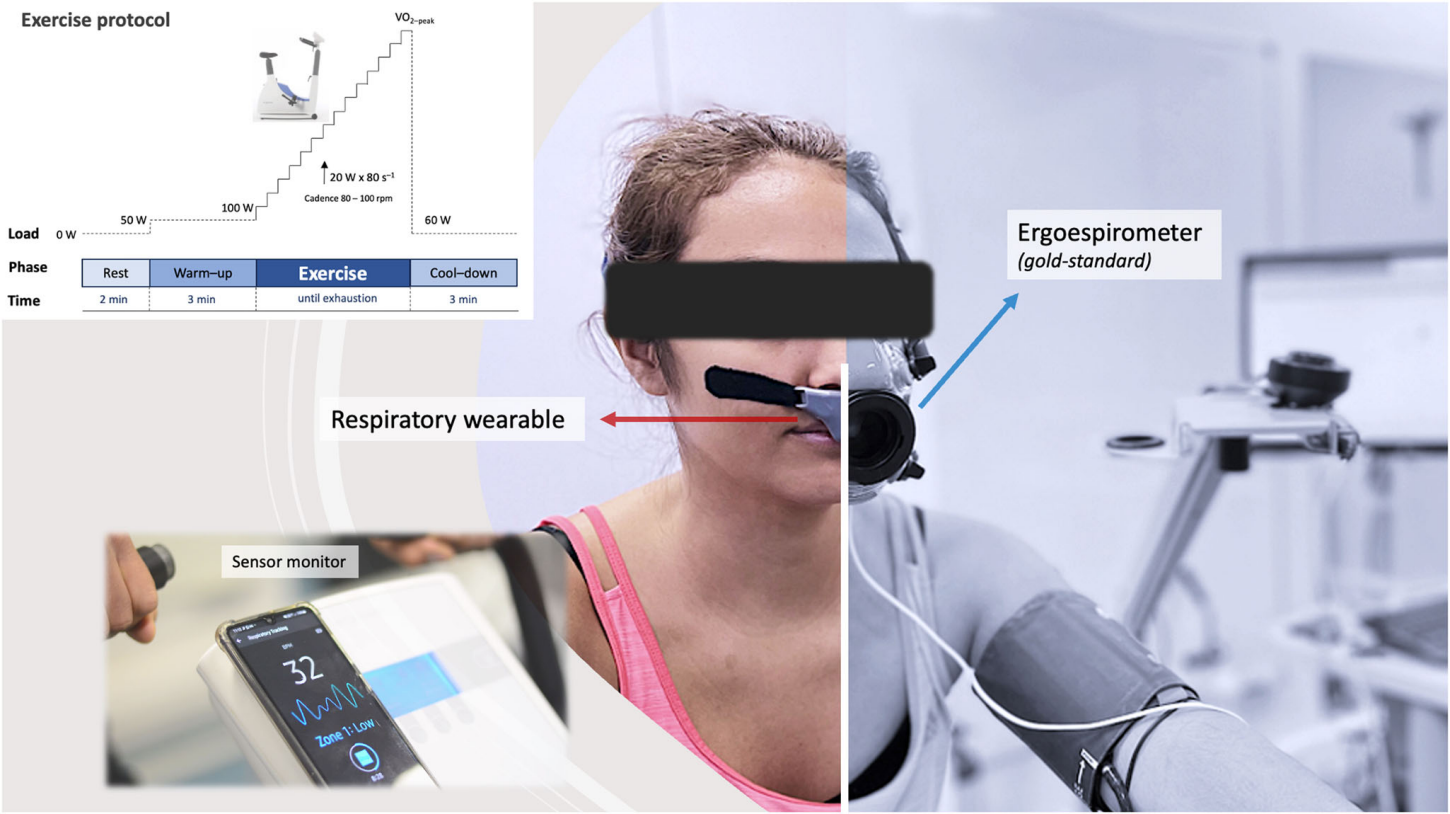
Experimental setup for wearable system validation. The ergospirometry face mask is installed on top of the sensor to obtain simultaneous measurements during tests. The wearable sensor broadcasts signals to a smartphone via Bluetooth, which records respiratory data for later analysis. The exercise protocol consisted in a ramp test with rest, warm-up, exercise, and cool-down stages. An informed consent for this image was obtained from the volunteer.

The pilot study on five subjects with repeated measurements showed that the placement of the wearable sensor inside the face mask does not significantly affect the time evolution of RR, tidal volume (VT), minute ventilation (MV), and VO_2_ measurements by the ergospirometer (Fig. 1, Supplementary Material). To assess the agreement between respiratory wearable and ergospirometry during exercise in the prediction of RR, we evaluated 17 healthy adults physically active who completed an incremental cycling exercise protocol (ramp test). Breath-by-breath RR was measured simultaneously by the wearable sensor and ergoespirometer (Fig. 1). Table 1 shows the participant characteristics and main variables registered during exercise protocol.

**Table 1:** Participant characteristics and main physiological variables during ramp tests (*n* = 17)

Age (year)	39.2 ± 9.7
Male/Female	10/7
Body weight (kg)	72.1 ± 9.5
Height (cm)	173.0 ± 6.5
BMI (kg • m^–2^)	23.9 ± 2.1
Cardiopulmonary exercise testing, CPET	
Aerobic or Ventilatory Threshold 1, VT1	
• %VO_2-peak_	62.1 ± 12.8
• Load_-peak_ (watts, W)	120 ± 5
• RR (bpm)	19 ± 4
• HR (bpm)	119 ± 15
• %HR_-theorical maximal_	66.1 ± 8.9
Anaerobic or Ventilatory Threshold 2, VT2	
• %VO_2-peak_	84.1 ± 4.9
• Load_-peak_ (watts, W)	209 ± 16
• RR (bpm)	32 ± 5
• HR (bpm)	156 ± 12
• %HR_-theorical maximal_	86.2 ± 6.5
Peak	
• %VO_2-peak_	100
• Load_-peak_ (watts, W)	242 ± 43
• RR (bpm)	44 ± 6
• HR (bpm)	167.0 ± 10.6
• %HR_-theorical maximal_	92.8 ± 6.2

Values are expressed as mean ± standard deviation.

*BMI* Body Mass Index, *%VO*_*2-peak*_ percent of peak oxygen consumption, *RR* respiratory rate, breaths per minute, *HR* heart rate, beats per minute, *%HR*_*-theoretical maximal*_ percent of maximal theoretical value of heart rate, according to formula [220-years].

**Fig. 2:**
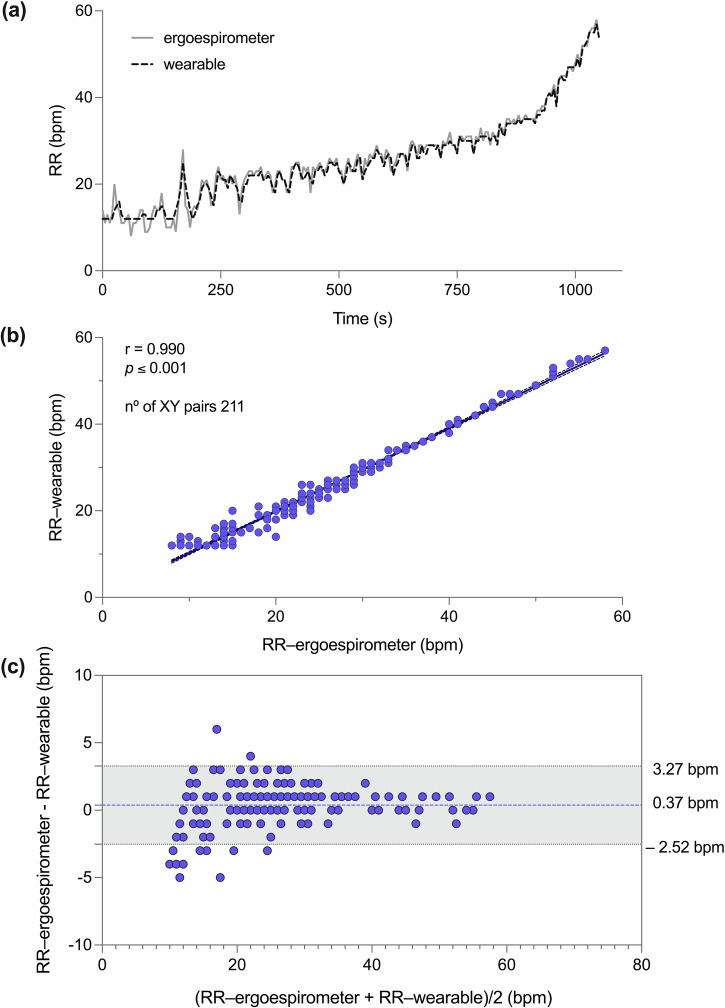
Wearable respiratory data and validation of RR against ergospirometry during a laboratory ramp for a representative subject (subject 8). **a** Visual comparison of RR time evolution, **b** Scatterplot with correlation analysis; **c** Bland–Altman plot.

A comparison between these two signals for one representative subject is shown in Fig. 2a, where we observe a high-visual agreement. For this subject, the correlation between the wearable and ergoespirometer was *very high* (*r* = 0.990, *p* ≤ 0.001, Fig. 2b). Bland–Altman (B&A) analysis resulted in a *low* bias of 0.37 breaths per minute (bpm) with a 95% confidence interval of (–2.52, 3.27) bpm, see Fig. 2c.

**Fig. 3:**
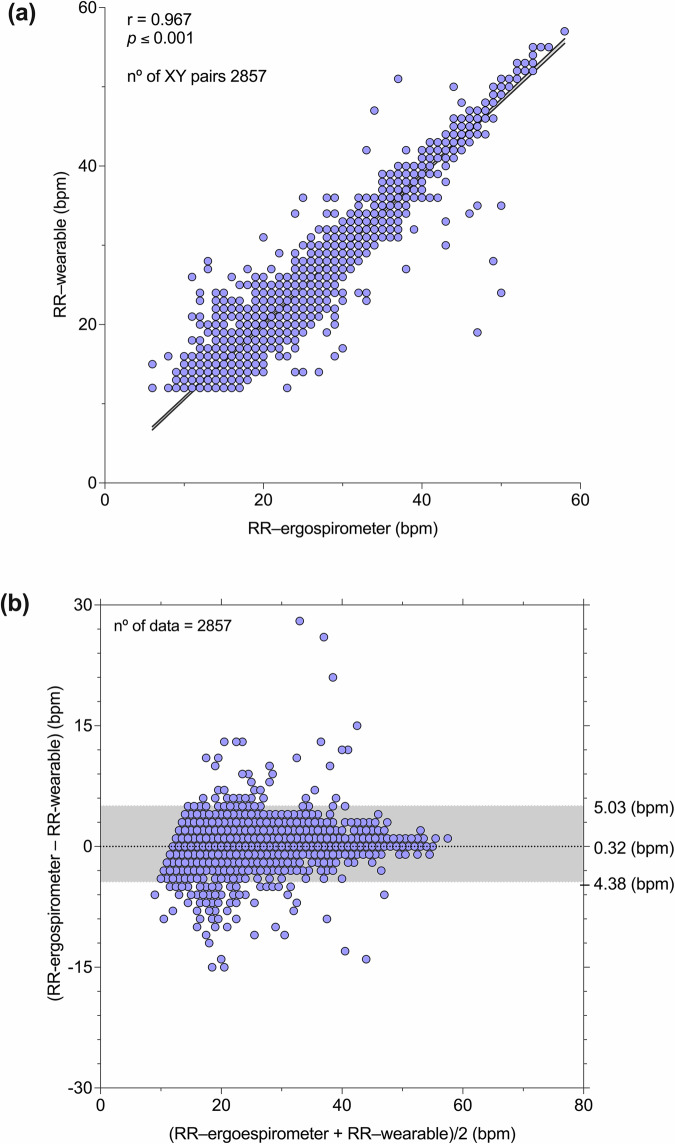
Comparison of RRs between respiratory wearable system and ergoespirometer during exercise in all participants. **a** Scatterplot with correlation analysis; **b** Bland–Altman plot.

Figure 3 shows the comparison of RRs between respiratory wearable and ergoespirometer during all exercise protocol of all participants (Fig. 3a); also- B&A analysis, showing a low bias of 0.32 bpm with 95% CI of (−4.32–5.03 bpm), see Fig. 3b.

Table 2 shows the bias, accuracy, and precision determined from B&A analysis for each subject in the study group. The group mean values of bias, precision, and accuracy were 0.33, 2.28, and 2.29 bpm, respectively. Bias in RR was found to be positive in most of the subjects. The Pearson correlation coefficient in the sample ranged from 0.91 to 0.98, with an average value of 0.95.

**Table 2:** Values of agreements between respiratory wearable and ergospirometry during exercise protocol in healthy adults physically active (*n* = 17)

Subject	Sex	# data points	Bias (bpm)	Bias 95% CI	Accuracy (bpm)	Precision (bpm)	*r*
1	M	199	0.11	[−4.99–5.20]	2.59	2.60	0.93
2	M	207	0.48	[−2.90–3.86]	1.78	1.72	0.98
3	M	165	0.42	[−3.65–4.49]	2.10	2.08	0.92
4	M	153	0.11	[−4.19–4.41]	2.19	2.19	0.97
5	M	233	0.14	[−4.62–4.90]	2.42	2.43	0.97
6	M	166	0.37	[–2.52–3.27]	1.51	1.48	0.99
7	F	210	0.50	[–2.84–3.83]	1.72	1.70	0.96
8	M	154	0.36	[–5.08–5.81]	2.76	2.78	0.94
9	M	208	–0.08	[–4.89–4.73]	2.41	2.45	0.92
10	M	198	0.52	[–3.64–4.68]	2.15	2.12	0.98
11	M	133	0.33	[–5.53–6.19]	2.98	2.99	0.91
12	F	160	0.16	[–4.85–5.17]	2.52	2.56	0.94
13	F	123	0.52	[–4.57–5.61]	2.62	2.60	0.94
14	F	156	0.64	[–2.56–3.85]	1.71	1.64	0.98
15	F	120	0.17	[–4.15–4.49]	2.19	2.20	0.96
16	F	127	0.37	[–5.81–6.55]	3.01	3.15	0.97
17	F	145	0.29	[–4.11–4.69]	2.16	2.25	0.97
mean ± SD		2857	0.33 ± 0.17		2.28 ± 0.44	2.29 ± 0.47	0.95 ± 0.02

*bpm* breaths per minute, *CI* confidence interval.

### Estimation and validation of ventilatory thresholds in laboratory conditions

**Fig. 4:**
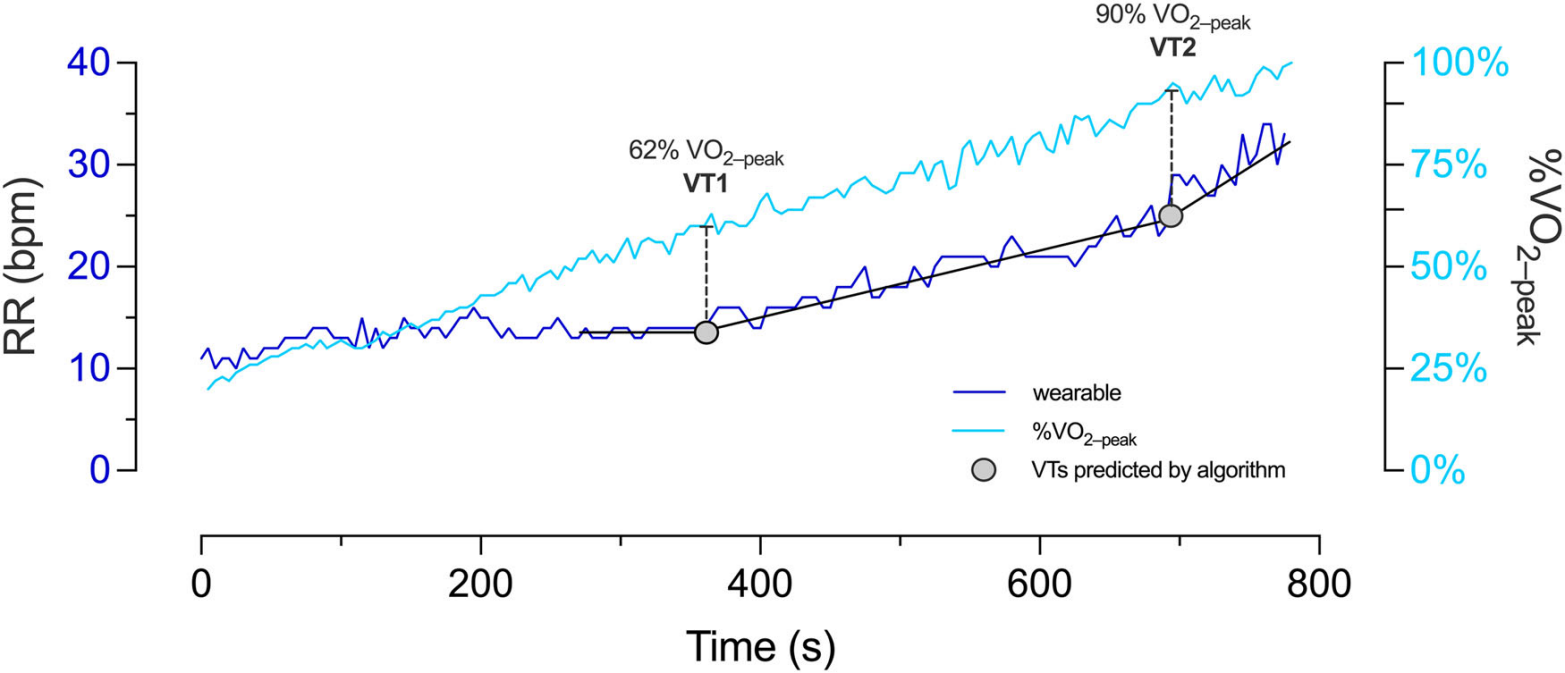
Comparison between wearable-system RR signal and time evolution of the rate of oxygen consumption during a laboratory ramp test in subject 4. Ventilatory thresholds predicted by the algorithm display a high agreement with those determined by expert physiologists.

Figure 4 shows the wearable RR evolution during a ramp test for a representative subject, the trilinear regression for the detection of respiratory breakpoints, and the VT1 and VT2 time points identified independently by two experienced physiologists who analyzed changes in MV and exercise-load during the protocol.

**Fig. 5:**
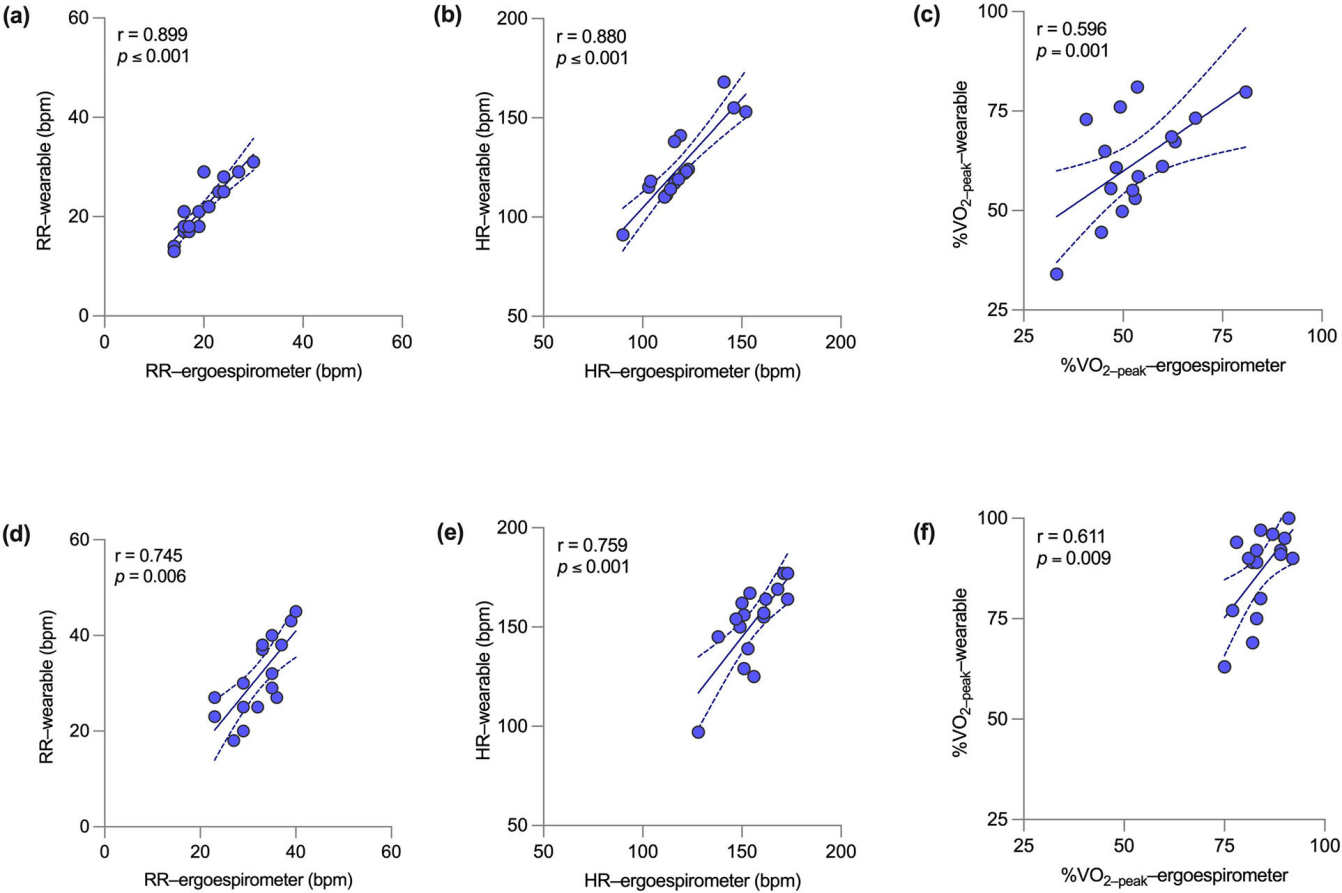
Correlation plots for the validation of VT wearable-system predictions. For VT1: (**a**) RR (bpm); (**b**) HR (bpm).; and (**c**) %VO_2–peak_. For VT2: (**d**) RR (bpm); (**e**) HR (bpm); and (**f**) %VO_2–peak_.

Respiratory breakpoints were evaluated in all subjects and were considered as predictors for the VTs. We compared these predictions with the VTs determined by expert physiologists in terms of the time-associated RR, heart rate (HR), and percentage of peak VO_2_ value (%VO_2-peak_). Figure 5 shows scatter plots reporting these comparisons. For the case of VT1, we observe a *high* correlation in terms of RR (*r* = 0.899, *p* ≤ 0.001) and HR (*r* = 0.880, *p* ≤ 0.001), and a *moderate* correlation in terms of %VO_2-peak_ (*r* = 0.596, *p* = 0.001). To VT2, the correlation was moderate for RR (*r* = 0.745, *p* ≤ 0.006) and HR (*r* = 0.759, *p* ≤ 0.001), and moderate *for* %VO_2-peak_ (*r* = 0.611, *p* = 0.009).

**Fig. 6:**
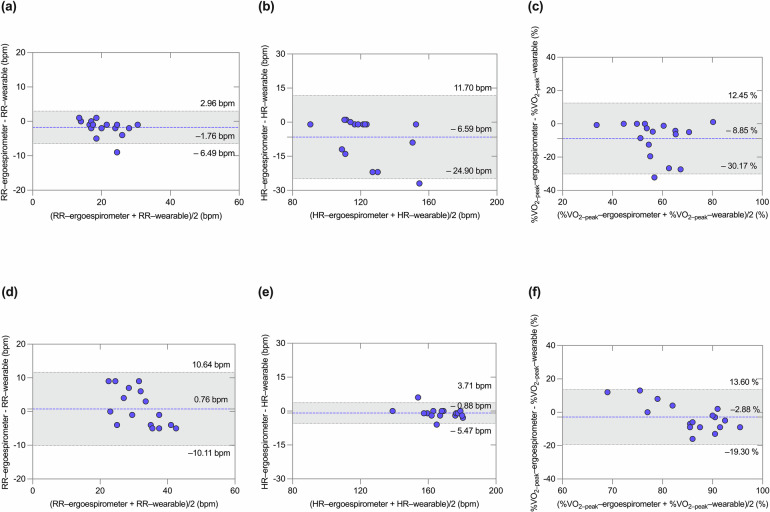
Bland–Altman plots showing the comparison between wearable-system and expert predictions of ventilatory thresholds. For VT1: (**a**) RR; (**b**) HR (bpm); and (**c**) %VO_2-peak_. For VT2: (**d**) RR; (**e**) HR (bpm); and (**f**) %VO_2-peak_.

Figure 6 shows the B&A analysis for the predictions of VT1 and VT2 in the sample group. For the case of VT1, the comparison of the wearable and ergoespirometer in terms of RR resulted in a bias of –1.76 bpm and limits of agreement (LoA) of from –6.49 to 2.96 bpm. For HR, the bias was –6.59 bpm and the LoA from –24.90 to 11.70 bpm. For %VO_2-peak_ measurements, the bias was –8.85% with LoA from –30.17 to 12.45%. When analyzing the prediction agreement for VT2, RR resulted in a bias of 0.76 bpm with LoA from –10.11 to 10.64 bpm, with all subject data inside LoA. To HR, the bias was –0.88 bpm with LoA from –5.47 to 3.71 bpm, and one participant was outside LoA; and finally, to %VO_2-peak_, the bias was –2.88% and all subjects data were inside LoA from –19.30 to 13.76%.

**Fig. 7:**
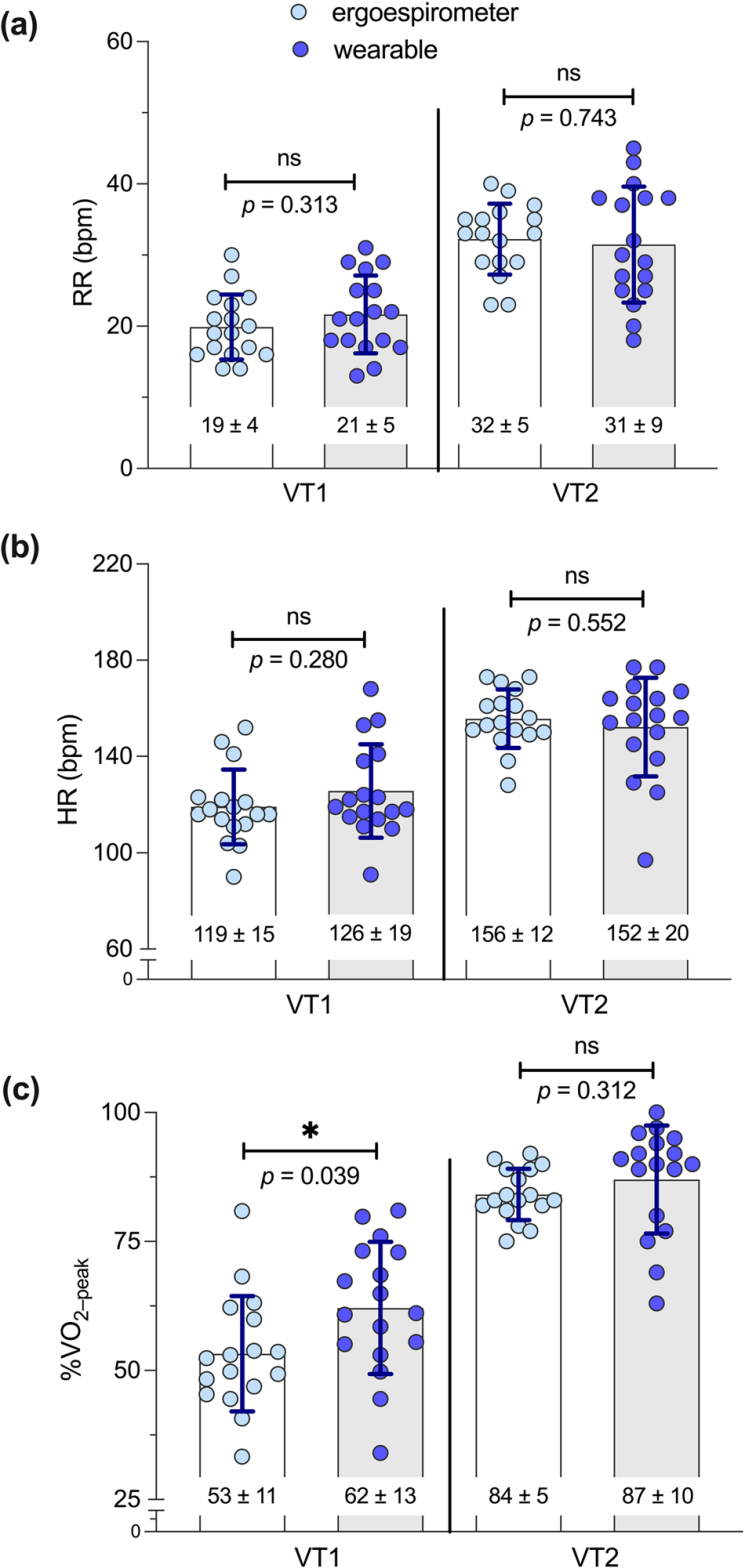
Group distributions of ventilatory thresholds determined during laboratory ramp tests by wearable system and gold standard. **a** RR; **b** HR; and **c** %VO_2–peak_.

Figure 7 shows a graphical comparison of RR, HR, and %VO_2-peak_ measured at VT1 and VT2 time points determined by experts from ergospirometry analysis and by the wearable system. No significant differences were found in these physiological parameters, except for %VO_2-peak_ at VT1.

### Study of ventilatory thresholds in a population of recreational athletes using the wearable system

**Fig. 8:**
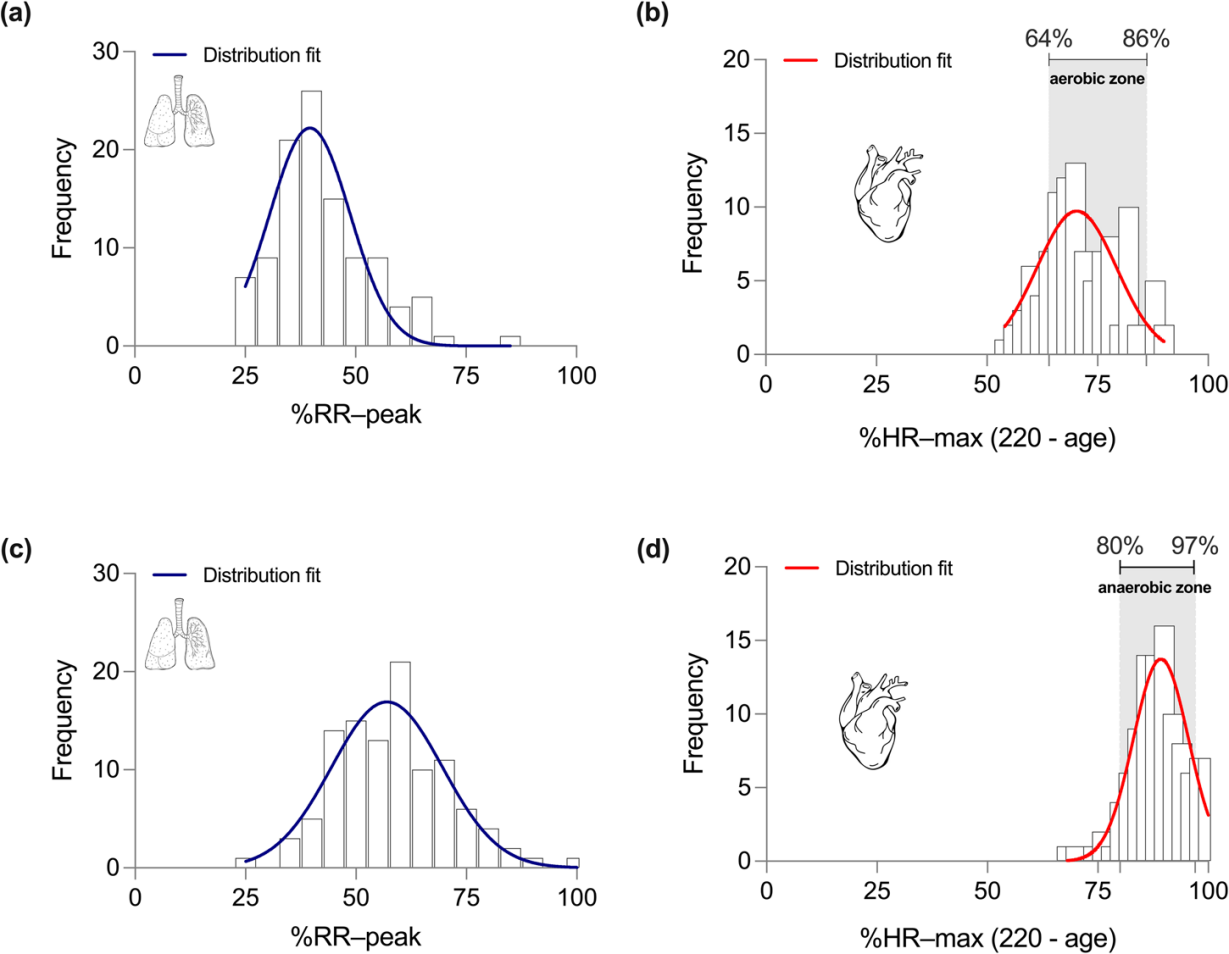
Population distribution of ventilatory thresholds in 107 recreational athletes evaluated with the respiratory wearable system. VTs were determined using the wearable system in ramp tests performed in training environments (outside the laboratory). For VT1, the distribution is shown in terms of (**a**) %RR–peak, and (**b**) %HR–max. For VT2, the distribution is shown in terms of (**c**) %RR–peak, and (**d**) %HR–max.

A total of 107 recreational athletes (20 females and 87 males, range age 31.1 ± 10.5 years) completed ramp tests outside the laboratory using our wearable system. Figure 8a, c shows the distributions of VT1 and VT2 expressed in terms of the percentage of the peak RR value achieved during the test (%RR–peak) for this population, respectively. The %RR–peak group values at VT1 and VT2 were 42.4 ± 12.6% and 58.3 ± 13.5%, respectively. Figure 8b, d shows the distributions of VT1 and VT2 expressed in terms of the percentage of the maximum theoretical heart rate (%HR–max.), respectively. The %HR–max group values at VT1 and VT2 were 71.9 ± 10.0% and 88.2 ± 8.4%, respectively.

## Discussion

Our wearable system achieves high accuracy in the continuous estimation of RR during incremental exercise, as evidenced when comparing the sensor predictions with ergospirometry airflow measurements (Fig. 2). RR wearable predictions were consistently higher than those measured by ergospirometry, as reflected by the positive bias in RR (Table 1). However, we note that in all subjects RR bias was always lower than 1 bpm, which can be considered almost negligible as 1 bpm represents the smallest unit employed in practical applications of RR monitoring. Interestingly, the performance metrics achieved in this study result in a similar mean error and dispersion to those obtained in healthy subjects breathing under resting conditions^26^. This result suggests that temperature-based sensors offer a robust approach for monitoring RR in a wide range of respiratory frequency and effort intensity (rest to maximal effort) on athletes training on a stationary bike. While several wearable sensors have been reported in the literature for monitoring RR in resting conditions^27,28^, and a few during low-stress walking activities^29^, systems that track RR during medium to high intensity exercise remain underexplored. One recent attempt considers acoustic sensors installed in a mouthguard during moderate exercise. The reported RMSD was 11.28% for a range of RR in 7–19 bpm as measured in 4 subjects under study. Higher activity intensity was associated with lower signal-to-noise ratio, which poses a challenge in the determination of RR from acoustic signals. A different approach is the use of face masks equipped with thermistors^30^. This mask, which fully covers the mouth and nose, funnels the airflow into a thermistor located at the front open end of the mask. A validation study in 10 male cyclists results in breath-by-breath RR overall bias and precision of –0.05 bpm and 3.37 bpm, respectively. Our overall RR metrics compare well with these values (Table 2) while providing a mask-free experience to the athlete that offers higher comfort during exercise.

Our wearable system and breakpoint algorithm achieve a high accuracy in the prediction of AT and RCT from ramp tests, with Pearson correlation values of 0.880 and 0.759, respectively, when measured in terms of HR (Fig. 5b,e). Further, the mean error for VT1 and VT2 measured in terms of respiratory rate are below 2 bpm (Fig. 6a, d), which is below the threshold considered relevant for medical applications^31^. In addition, no significant differences between the *gold-standard* method and the wearable system predictions were found when comparing group mean values of VTs (Fig. 7). Many of these performance metrics are in the range of those achieved by ECG-based solutions^22,23^ for VT1 and by NIRS-based technologies for VT2^24,32^, with the convenience of providing both VTs using only one wearable technology. A key advantage of estimating VT1 and VT2 from respiratory signals is the direct and well-established connection between metabolic changes and non-linear changes in respiratory flow during exercise^33^, which are not necessarily manifested by the cardiac or muscular systems^9^. Based on these grounds, we anticipate that our system can be predictive not only on the population considered in this study, but their reach can extend to other cohorts in non-controlled environmental conditions that may affect VTs^34–36^. This feature may prove advantageous when compared to algorithms that rely on large amounts of labeled data, such as neural networks or other deep learning algorithms, which may not be available for specific populations or environmental conditions, where little or no data is available for algorithm training.

One promising application of the respiratory wearable system demonstrated in this work is the evaluation of ventilatory thresholds in a large population outside of the laboratory. While the validation of individual estimations in this setting is unfeasible, the empirical population distributions of VT1 and VT2 tested with our wearable system can be compared to previous laboratory studies on large groups of volunteers. A recent study in a group of 100 healthy adults with varying levels of fitness showed that the sample average aerobic and anaerobic lactate thresholds were found at 74.9% and 89.0% of the maximum heart rate^16^. These values compare extremely well with those found for average values of VT1 and VT2 in our study: 72.4% and 88.7% of the maximum heart rate, respectively. Further, unimodal distributions around these values are observed in both studies. In addition to these heart-rate population metrics, our work provides distributions and average values of VT1 and VT2 expressed in terms of the peak respiratory rate, see Fig. 8. We note that the percentage of peak respiratory rate highly correlates with the rating perceived exertion index, which is a widely used index used for the subjective assessment of physical intensity^37^. Thus, monitoring RR during training may provide an objective measurement of perceived exertion for classifying physical intensity, particularly if ventilatory thresholds can be determined in terms of RR.

This work represents the first validation of a wearable system for ventilatory threshold evaluation, and as such it offers several opportunities for future developments and applications. First, we note that while the mean error in estimating VT1 and VT2 was relatively low when measured in terms of RR and HR, the bias achieved in terms of %VO_2-peak_ may be large for certain applications. Further, the dispersion in these parameters, measured in terms of precision and limits of agreement, can be higher than that reported by other solutions^22,32^. Future developments of the wearable system could reduce this variability by developing a minute-ventilation estimator based on the sensor signal accurate for exercise conditions and then performing a breakpoint analysis on such volumetric data^9^. Despite this potential improvement, we note that dispersion in VTs determination can be high in the gold-standard method, as significant systematic differences among expert evaluators using the visual method have been reported in the literature^38^. Second, we remark that the proposed VTs detection algorithm relies on a triphasic exercise model of MV during ramp tests^17^. While this physiological model applies to healthy trained subjects, it may not be experienced by undertrained participants or patients with chronic cardiorespiratory diseases^19,39–41^. In these low-aerobic capacity subjects, typically VT2 is not attained during ramp tests^42^. Future efforts may take this modified intensity model into consideration to develop enhanced algorithms for the determination of VT1 using the wearable system. Third, while our population study was performed outside the laboratory, it typically consisted of indoor ramp tests in gymnasiums and home setups. One interesting opportunity is the assessment of ventilatory thresholds in adverse environmental conditions, such as high altitude or extreme temperatures, where standard variables like heart rate have shown to lose accuracy in estimating exercise intensity^20,21^. This aspect is particularly useful in sports such as climbing, cycling, mountaineers, triathletes, and trail runners, where the extensive competition time and extreme environmental conditions require accurate estimation of effort intensity to achieve optimal sports performance. Finally, future efforts should further explore the evaluation of ventilatory thresholds for clinical purposes. One such opportunity applies to physical rehabilitation programs, where the accurate determination of effort intensity is critical in the recovery of pulmonary functional capacity in survivors of severe COVID-19 where oxygen uptake and physiological responses to exercise are impaired^43,44^. An adequate exercise prescription, based in determination of VT1 and VT2 as markers of effort intensity also becomes critical in patients undergoing pharmacological treatment, where heart rate is affected (e.g., negative chronotropes drugs or beta-blockers), and therefore cannot be used to classify exercise intensity^45–47^. The same applies to patients with significant peripheral atrophy or with high adipose tissue thickness where non-invasive monitoring of muscle oxygenation loses accuracy^48,49^, or patients experiencing exacerbated symptoms of dyspnea and muscular fatigue^50,51^.

## Methods

### Wearable respiratory sensor and ventilatory-threshold detection algorithm

**Fig. 9:**
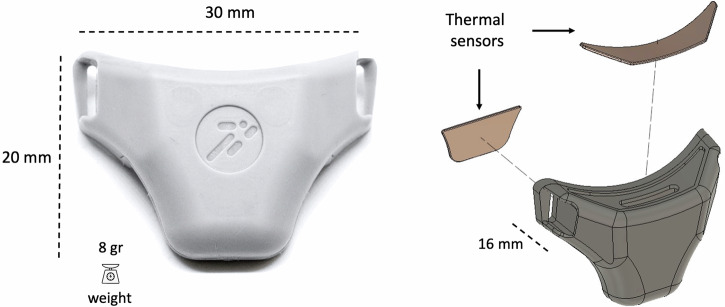
Respiratory wearable sensor: device dimensions and sensing components.

The respiratory wearable sensor is built upon a wearable temperature-based sensor previously employed in predicting respiratory rate (RR) and MV in subjects at rest^26,52^. It consists of a small and lightweight case (length 30 mm, width 16 mm, height 20 mm; weight 8 g) with two thermal sensors directly placed under the nose and in front of the mouth, see Fig. 9.

Nasal and oral sensors measure temperature changes that result from nasal and oral airflow impinging the sensor plates, respectively. These temperature signals are broadcasted via Bluetooth to an external device (smartphone) which registers these signals for offline analysis. Temperature time series are analyzed using a mean-cross algorithm to estimate breath-by-breath RR as detailed in a previous contribution^26^. The breath-by-breath RR dataset is then interpolated every 0.04 s to create a uniformly spaced time series with sampling frequency of 25 Hz. This RR time series is exported from the smartphone for further offline analysis.

During offline analysis, and to enable a fair comparison with ergospirometry data, the raw RR time series obtained from the wearable system for each subject was processed using a moving average filter (window size of 1 s), from which a subsampled time series with RR measurements every 5 s was created. This processed RR time series was employed in the validation of RR predictions and in the estimation of VTs.

The determination of VTs was performed by detecting abrupt changes in the respiratory flow pattern during a ramp test, measured as breakpoints in the RR time evolution^33^. To this end, the RR time series is first filtered using a third order SavGol filter (SciPy v1.10.1) with a window size of 30 s to remove short-term fluctuations. This choice of filter parameters allows for capturing inflection points inside the window size, as well as ensuring that enough data points are approximated to prevent overfitting. To detect breakpoints, we performed a trilinear segmented regression using the filtered RR time series. We consider the following regression function:

1 $$R{R}_{{reg}}\left(t\right)=\left\{\begin{array}{cc}{a}_{0}+{b}_{0}t & 0\, <\, t\le {t}_{1}\\ {a}_{1}+{b}_{1}(t-{t}_{1}) & {t}_{1} < t\le {t}_{2}\\ {a}_{2}+{b}_{2}(t-{t}_{2}) & {t}_{2} < t\le T\end{array}\right.$$

where *T* is the final time at which the ramp test stops, and $${a}_{0},{b}_{0},{a}_{1},{b}_{1},{t}_{1},{a}_{2},{b}_{2},\,{t}_{2}$$ are parameters to be determined. In particular, pairs $$({t}_{1},\,{a}_{1})$$ and ($${t}_{2},{a}_{2})$$ in Eq. (1) represent the time and RR tuples where breakpoints are located, which we use to predict VT1 and VT2. Assuming the regression function is continuous at breakpoints located at *t*_1_ and *t*_2_, we find expressions for *a*_1_ and *a*_2_ in terms of an independent set of parameters given by $${a}_{0},{b}_{0},\,{b}_{1},{t}_{1},{b}_{2},\,{t}_{2}$$. This last set of unknown parameters is determined by solving a least-squares fit of the regression function to the filtered RR time series. This piecewise regression procedure is implemented by the Python pwlf library (v2.2.1), which we used in our calculations.

### Laboratory cardiopulmonary exercise tests in healthy subjects

Seventeen (*n* = 17) healthy adults (age = 19–44 years) were recruited in a non-probability convenience sampling through social network advertising to perform an incremental CPET to study ventilatory thresholds. Inclusion criteria were physically active (≥150 min of moderate or ≥75 min of vigorous physical activity by week), with normal body mass index (20–25 kg · m^–2^). Exclusion criteria were history of respiratory, cardiovascular, metabolic, musculoskeletal, or neoplastic diseases or without any infectious or inflammatory process at least 2 weeks prior to the beginning of the study. All participants were informed orally and verbally of the purpose, protocol, and procedures before informed consent was obtained. This study was conducted in accordance with the Declaration of Helsinki and approved by the Ethics Committee of Pontificia Universidad Católica de Chile (Institutional Review Board, protocol number ID210125002, date of approval: April 8, 2021). Informed consents were obtained from all participants in the study. The authors affirm that human research participants provided informed consent, for publication of the images in Fig. 1.

All experiments were carried out in the Laboratory of Exercise Physiology at Alemana Sport, Santiago, Chile, using a commercial CPET bicycle ergometer (MasterScreen™ CPX, Jaeger®, Germany). Procedures were performed under constant laboratory environmental conditions (temperature 20 ± 2 °C; relative humidity, 40 ± 2%) and within a similar time frame (from 9:00 to 14:00 h). Participants were asked to avoid physical activities for 24 h before the measurements and to avoid alcohol, caffeine, and other stimulants and food for at least 3 h prior to the evaluations.

Before the tests, subjects were equipped with the respiratory wearable sensor and with an ergospirometry facial mask. Exhaled gases (VO_2_ and VCO_2_) and ventilatory variables (VE, TV, and RR) were measured using an airflow turbine connected to the facial mask under standard pressure dry air temperature (SPTD). Simultaneously, the respiratory wearable sensor registered continuous RR throughout the test. Heart rate and pulse oxygen saturation were also continuously monitored during exercise using a cardiothoracic band and an oximeter, respectively. The exercise protocol consisted of an initial register of 60 s to allow for sensor synchronization, followed by a 2-min baseline phase (0 watts (W), rest-phase), followed by 3-min of warm-up period at 50-W, and then followed by the exercise phase with 80 s intervals and initial load of 100-W, see Fig. 1 for a schematic. The load was increased by 20-W after each interval. Participants were requested to maintain a cadence between 80 and 100 rpm during protocol. A cool-down phase of 3-min at 60-W was performed during the final stage of the test (Fig. 1).

To assess the effect of the wearable sensor in ergospirometry measurements, a pilot study considering five volunteers was carried out, where maximal tests were conducted twice on the same subject, with and without the wearable sensor. The comparison of the RR, VT, VE, and VO2 time evolution between these cases is reported in Supplementary Fig. 1.

Using the ventilatory variables and exhaled gases values recorded by the ergoespirometer during exercise protocol, two blinded experienced physiologist researchers determined VT1 and VT2. In case of discrepancy, the opinion of a third blinded research was possible, accepting as the definite criterion that point at which at least two evaluators agreed^53^. Determination of VTs were based on the loss of linearity between VE and load of each interval of load-work during protocol. Values correspond to the mean of the last 30-s of each interval load^15,17,54^. For elucidating this aim about if changes of RR-wearable during exercise could identify VTs, were compared the values of HR and %VO_2-peak_ at which RRs´ trends registered by respiratory wearable determined VTs with those registered at which VTs were determined by exhaled gases analyses using ergospirometer.

### Ramp tests using the wearable system outside the laboratory

The respiratory wearable system was used by 107 recreational cyclists (20 women) during ramp tests on bicycles connected to commercial smart trainers. All tests were conducted in training environments under uncontrolled conditions (e.g., gyms or at home). Tests were conducted using a virtual cycling platform (Zwift Inc., California, USA) with bikes connected to an electronically braked cycle ergometer indoor trainer device (KICKR™, Wahoo Fitness, Atlanta, GA, USA). The protocol consisted of 1-min at resting phase where the wearable was turn on, 3-min of warm-up phase (50-W), and exercise phase that consists of intervals of 80 s, starting at 100 W and increased 20-W by each interval period, until voluntary exhaustion. At the end, a cool-down phase of 3-min at 60-W. The directed cadence was between 80 to 100 rpm. Each subject was assisted remotely in the installation of the respiratory wearable sensor and in connecting the sensor to the app on a smartphone. After the test, the RR signals from the wearable sensor and cardiac and power data from the virtual cycling platform were collected for offline analysis. The resulting estimates of VT1 and VT2 were computed in terms of HR and RR at the associated time instants and was normalized by the estimated maximum heart rate (equal to 220 minus age) and peak RR, respectively.

### Validation metrics

We performed Bland–Altmann and correlation analyses to assess the agreement and association between the wearable system predictions and the *gold-standard* values. To compare our results with previously reported metrics, we also evaluated the performance of the wearable-system predictions in terms of bias, precision, accuracy, and the Pearson correlation coefficient^52^. In brief, assuming there are *N* subjects, for the i-th subject we define ${\hat{X}}_{i}$ and *X*_*i*_ as the predicted and *gold-standard* values, respectively. Then, absolute bias, precision, and accuracy are determined as follows:

2 $${Bias}=\frac{1}{N}\mathop{\sum }\limits_{i=1}^{N}({\hat{X}}_{i}-{X}_{i})$$

3 $${Precision}=\sqrt{\frac{1}{N}\mathop{\sum }\limits_{i=1}^{N}{{(\hat{X}}_{i}-{X}_{i}-{Bias})}^{2}}$$

4 $${Accuracy}=\sqrt{\frac{1}{N}\mathop{\sum }\limits_{i=1}^{N}{{(\hat{X}}_{i}-{X}_{i})}^{2}}$$

For the analysis of time series data in a single individual (i.e., validation of RR), index *i* in Eqs. (2)–(4) denotes a time instant and *N* denotes the total number of measurements in that individual. From their definitions, we interpret bias and precision as the average error and standard deviation of the error of the predicted values, respectively. Further, accuracy can be related to the root-mean-square error of the prediction.

For the validation of VTs, we determined the RR, HR, and %VO_2-peak_ values at VT1 and VT2 and performed Bland–Altman and correlation analyses based on these physiological parameters.

### Statistical analysis

For intergroup comparison, normality was assessed by the Shapiro–Wilk test, and mean differences were tested using the unpaired *t*-test. Numerical results are expressed in terms of mean and standard deviation.

### Supplementary information

Supplementary Information

## Data Availability

The datasets generated and/or analyzed during the current study are not publicly available due to subject privacy protection but are available from the corresponding author upon reasonable request.

## Code availability

The underlying code for this study is not publicly available but may be made available to qualified researchers on reasonable request from the corresponding author.
