# Supplementary material for: Estimation of ventilatory thresholds during exercise using respiratory wearable sensors
Source: NPJ Digit Med. 2024 Jul 26;7:198. doi: 10.1038/s41746-024-01191-9 (PMC11282229; doi:10.1038/s41746-024-01191-9)
Supplement: Supplementary file 1 — Supplementary Information [file 41746_2024_1191_MOESM1_ESM.pdf]

Supplementary Information: Estimation of ventilatory thresholds during exercise using respiratory wearable sensors

Felipe Contreras–Briceño; Jorge Cancino; Maximiliano Espinosa–Ramírez; Gonzalo Fernández; Vader Johnson; Daniel E. Hurtado

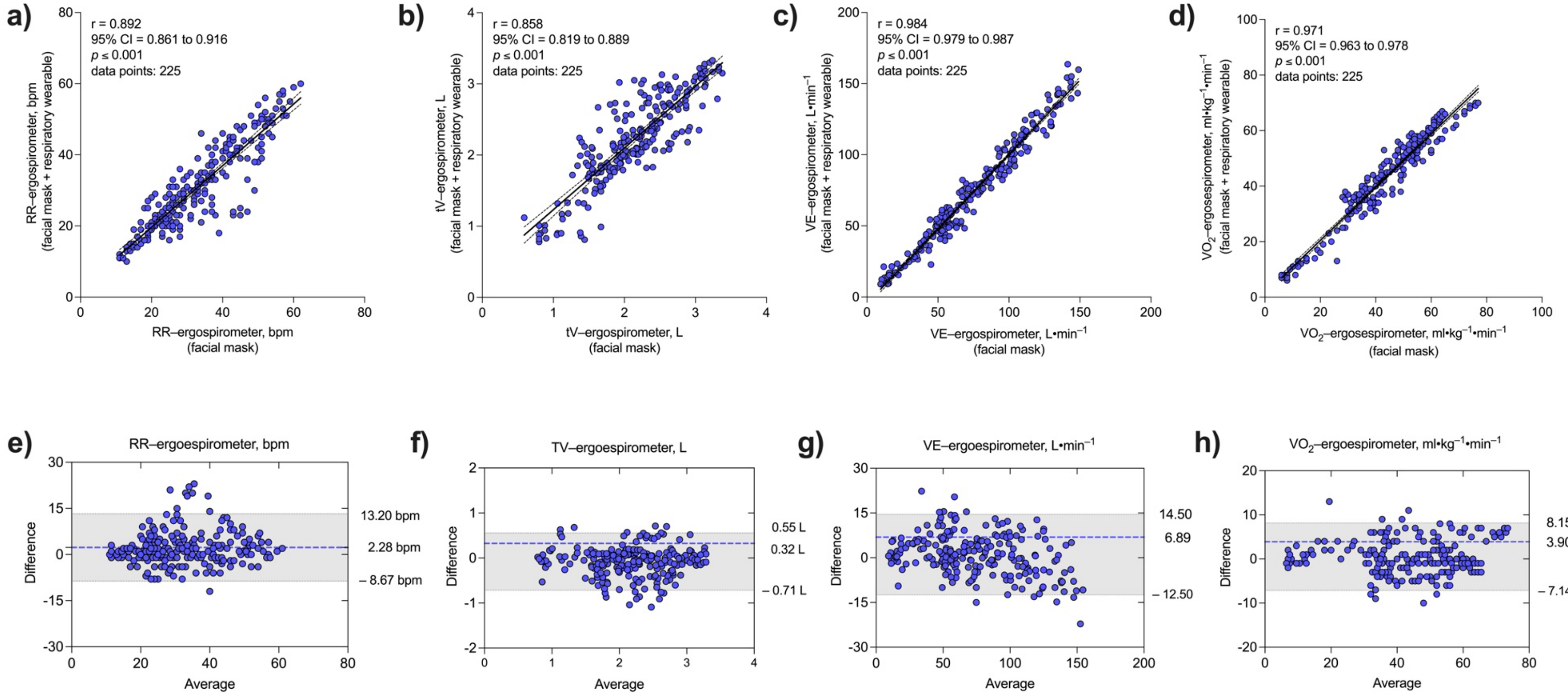

Supplementary Figure 1. Effect of the wearable sensor on metabolic and ventilatory measurements during ergospirometry studies. Comparison of the time evolution of respiratory rate, (RR), tidal volume (TV), and lung ventilation (VE)) and oxygen consumption (VO_2) registered by ergospirometry with and without the respiratory wearable inside the facial mask during an incremental exercise test performed by five healthy adult volunteers. (a), (b), (c), and (d) Scatterplots with correlation analysis; (e), (f), (g), and (h) Bland–Altman plots.
